# Evaluation of Protective Equipment Used Among Motorbike Riders

**Published:** 2018-05-18

**Authors:** Roxanne Stiles, Clint Benge, P.J. Stiles, Fanglong Dong, Jeanette Ward, Elizabeth Ablah, James M. Haan

**Affiliations:** 1 Department of Surgery, University of Kansas School of Medicine-Wichita, Wichita, Kansas; 4 Department of Preventive Medicine and Public Health, University of Kansas School of Medicine-Wichita, Wichita, Kansas; 2 Western University of Health Sciences, Pomona, California; 3 Chandler Regional Medical Center, Chandler, Arizona; 5 Via Christi Hospital, Wichita, KS

**Keywords:** off-road motor vehicles, personal protective equipment, trauma, safety, Kansas

## Abstract

**Introduction:**

This study compared outcomes between patients injured at a motorbike track, which requires riders to follow safety equipment guidelines, and those involved in recreational riding where safety equipment usage is voluntary.

**Methods:**

A retrospective review was conducted of all patients presenting with motorbike-related injuries at an American College of Surgeons verified level-I trauma center between January 1, 2009 and December 31, 2013. Data collected included demographics, injury details, safety equipment use, hospitalization details, and discharge disposition. Comparisons were made regarding protective equipment usage.

**Results:**

Among the 115 patients admitted, more than half (54.8%, n = 63) were injured on a motorbike track, and 45.2% (n = 52) were injured in a recreational setting. The majority of patients were male (93.9%), Caucasian (97.4%), and between the ages of 18 to 54 (64.4%). Helmet usage was higher among track riders (95.2%, n = 60) than recreational riders (46.2%, n = 24, p < 0.0001). Comparisons of injury severity and outcomes between those who wore protective equipment and those who did not were not significant.

**Conclusion:**

Even though track riders wore protective equipment more than recreational riders, there was no difference between the groups regarding injury severity or hospital outcomes. These results suggested that motocross riders should not rely on protective equipment as the only measure of injury prevention.

## INTRODUCTION

Motocross is a high-risk endurance sport where off-road motorbikes (or dirt bikes) are put through challenging obstacles at high rates of speed.^1^–^11^ This sport is particularly popular among males younger than 30 years, although in the United States (U.S.) children as young as four can compete, and the sport has begun to attract family participation.^1^–^7^ Organized motocross events can occur in regulated arenas, but recreational motorbike use on unregulated private property is also popular.^1^–^16^ Recent data from the National Electronic Surveillance System-All Injury Program (NEISS-AIP) from 2001–2004 indicated that 20% of off-road motorcyclists (≤19 years) treated for non-fatal injuries were from motocross areas, the remaining were from other off-road locations.^11^

The majority of injuries sustained by motocross participants include minor contusions and lacerations, however, more serious injuries such as extremity fractures and head injuries are also common.^1^–^12^ In the U.S., motocross has the fourth highest incidence of head and neck injuries suffered by athletes who participate in extreme sports.^17^ Recreational off-road motorbike riders experience similar injuries as riders in regulated events, yet these riders are also less likely to wear protective equipment.^12^–^16^

For racing in American Motocross Association sanctioned events, a full-face helmet is required which conforms to recognized Snell M2010 or Department of Transportation standards.^18^ Additional safety equipment that also may be required includes shatterproof goggles, body armor, protective pants and long-sleeve jerseys, kneepads or braces, gloves, and boots. However, for recreational off-road activities, most states do not have safety regulations or requirements.^18^,^19^ Consequently, the use of protective equipment is voluntary. Currently, Kansas has no restrictions on operator age, licensure requirements, helmet or eye protection regulations, or mandatory educational programs to operate motorbikes off-road.^19^

In the current study, outcomes associated with motorbike crashes were examined. Also, the types of safety equipment worn at the time of injury were identified. This study compared outcomes between patients injured at a motorbike track (who were more likely to have been required to follow equipment safety guidelines) and patients injured during recreational motorbike activities (where safety equipment usage is voluntary) to determine if safety equipment use in motorbike activities makes a difference in patient outcomes.

## METHODS

A five-year retrospective chart review was conducted of all patients admitted with injuries sustained while operating a motorbike between January 1, 2009 and December 31, 2013. Eligible patients were identified through the trauma registry of an American College of Surgeons verified level-I trauma center. Patient’s charts were reviewed to distinguish between an off-road motorbike and a standard motorcycle crash and to identify if the crash occurred at a local motocross track (TR) or on private property (RR). Recreational crashes were defined as those that occurred on private unpaved or other road surfaces. Track riders were defined as those who sustained an injury while riding on one of several local motocross tracks. Data collected included patient demographics, injury severity score (ISS), and crash details (crash type, location, and protective equipment worn). Details of the patient’s hospitalization included hospital length of stay (HLOS), intensive care unit length of stay (ICU LOS), ventilator days, discharge disposition, and mortality.

All statistical analyses were conducted using SAS software for Windows, version 9.3 (Cary, North Carolina). Descriptive analyses were presented as means and standard deviations or median and interquartile range (IQR) for continuous variables, if the sample size was too small, along with frequencies and proportions for categorical variables. Continuous variables were compared using t-tests, and categorical data were compared using Chi-square analysis or the Fisher’s exact test when appropriate. Patients were stratified by the crash location (track rider vs. recreational rider) and comparisons were made regarding protective equipment usage and hospital outcomes. In addition, a sub-analysis was conducted comparing the adult rider population (> 17 years of age) with the pediatric rider population (0–17 years of age). All tests were two-sided, and a p value < 0.05 was considered significant. This study was approved for implementation by the appropriate Institutional Review Boards.

## RESULTS

A total of 115 patients were admitted for motorbike-related injuries. Most were male (93.9%, n = 108) and Caucasian (97.4%, n = 112) with an average age of 26.2 ± 13.4 years and ISS of 7.5 ± 6.1. Seventy-four patients (64.4%) were aged 18 – 54 and 31.3% (n = 36) were considered pediatric (Table 1). More than half of patients were injured on a motorbike track (54.8%, n = 63) and 45.2% (n = 52) were injured in a recreational setting. Almost one quarter (23.5%, n = 27) were admitted into the ICU, and 5.2% (n = 6) were on a ventilator. Most patients (90.4%, n = 104) were discharged home. An adult recreational rider with no protective equipment died of his injuries. There was no statistically significant difference between the study groups for demographics, hospital outcomes, and discharge destination.

Comparison of protective equipment usage between the groups is presented in Table 2. The most common safety equipment reported for the total population was a helmet (73.0%, n = 84). Track riders were more likely to wear a helmet (95.2% vs 46.2%, p < 0.0001) and protective clothing (76.2% vs 15.4%, p < 0.0001) compared to recreational riders. No other protective equipment usage was documented in the RR group.

Comparisons based on protective equipment status and crash location are presented in Table 3. Regardless of crash location, those with documented protective equipment had the highest average ISS with the TR population being statistically significant. Patient HLOS varied among the RR and TR populations. For instance, RR without documented protective equipment had the longest HLOS (3.0 ± 3.8) while TR patients with protective equipment had the longest HLOS (3.2 ± 4.5), ICU LOS (median = 1, IQR = [1, 3]) and most ventilation days (median = 13, IQR = [3, 20]). However, there were no differences based on age, HLOS, ICU length of stay, and ventilation days.

A sub-population comparison among adult and pediatric riders demonstrated that most pediatric riders wore protective equipment and experienced a lower average ISS than the adult riders. Among the RR population who wore protective equipment, adults had the highest average ISS (10.0 ± 8.9) while pediatric riders had the lowest average ISS (4.7 ± 1.9) and the shortest average HLOS (1.8 ± 1.8). Among the TR population, adult riders with protective equipment had the highest average ISS (9.3 ± 6.9), and the longest average HLOS (3.8 ± 5.4). However, these results were not statistically significant (not shown).

## DISCUSSION

Motorbike trauma patients in this study were most likely to be adults, Caucasian, and male, with more overall crashes occurring at motorbike tracks. The form of safety equipment most commonly worn by both groups was a helmet. However, recreational riders were less likely to wear helmets compared to riders injured on motorbike tracks, where safety equipment requirements are enforced. This finding was not surprising given evidence that without mandatory helmet laws, helmets are worn less frequently.^20^,^21^

Overall, most motorbike injuries in the current study were not severe. When compared to previous studies, the overall current RR population was injured less severely,^12^–^15^ however, the TR population was injured more severely.^3^,^7^,^12^ Regarding patient age, the most severely injured riders in the current study were adult riders who wore protective equipment, regardless of crash location. No severe injuries were found in the pediatric population, with RR pediatric riders who wore protective equipment having the lowest average ISS. Possible reasons for why the adult population had higher ISS than the pediatric population could be related to the nature of the crash, having a larger motorbike engine size, or participating in more risky behaviors.

Although the majority of the TR population in the current study wore protective equipment and the RR population did not, there were no statistically significant differences between the groups regarding injury severity and hospital outcomes. These results are consistent with various adult and pediatric motocross studies which demonstrated that despite protective equipment use motorbike riders still experienced a high rate of injuries.^1^,^2^,^4^,^5^,^7^,^8^,^12^ For instance, a pediatric motocross study found 50% of patients sustained concussions and 69% orthopedic injuries, even though all patients wore full protective gear (helmets, goggles, protective pants, long-sleeve jersey, and boots).^1^

Based on these findings, other injury reduction measures such as focusing on risk factors that may be associated with increased injury rates are needed. Risk factors that may increase the chance of being injured while participating in motorbike activities include rider experience, hours of training, being under the influence of alcohol or drugs, size of motorbike engine, and speed and nature of the crash. For example, Colburn et al.^16^ illustrated that jumping during motocross activities results in higher injury severity. This may explain why there were no differences between the two groups in our study since those injured on a track may have involved more jumps than those injured during recreational riding.

In addition, collisions and being run over by other riders may be more common for track riders than for recreational riders due to the proximity of other riders. In fact, Larson et al.^4^ indicated that many severe injuries were related to collisions with other riders or as the result of being run over. However, due to the retrospective nature of this study, this information was not obtained. To understand the interplay between hospital outcomes and injuries resulting from motocross injuries, prospective studies are needed to define the circumstances that are involved in motocross crashes, including details on crash terrain and track design. Rider characteristics such as risk-taking behavior, rider experience and looking to see if riders with protective equipment are more likely to be involved in risk-taking behaviors than riders without protective equipment are also important.

In the current study, it would appear that protective equipment use during motocross activities is not warranted due to the lack of differences between those who did and did not wear protective equipment. However, the majority of injuries were not severe, and the mortality rate was low (0.8%) indicating that protective equipment use may have prevented more serious injuries. Further, we were unable to delineate critical descriptive data related to the rider’s level of experience or characteristics of either the vehicle involved or the location where the accident occurred.

Helmet use is recommended for protection against severe head injuries and mortality.^15^–^17^ Additional gear such as extremity protection and chest plates are encouraged due to the high rate of fractures and thoracic injuries.^1^–^12^,^14^ Further, age restrictions and safety course/certification for minors, focusing on course designs, and requiring all participants to have protective gear fitted by a professional, should be implemented for all motorbike participants.^2^,^9^

There are limitations to this study. First, this study was retrospective and conducted at a single facility. The study was limited by a relatively small sample size and by the lack of consistent reporting of safety equipment in patient charts. Also, it was difficult to differentiate the specific type of two-wheeled vehicle utilized by the rider (e.g., a moped, motorcycle, motocross/recreational motorbike) based on patient charts. In addition, with the possibility of injured riders being admitted to another facility or being treated at the scene, not all motorbike- related injuries were represented in this study. Finally, it was difficult to distinguish from patient records whether participants injured on tracks were participating in sport versus riding recreationally.

## CONCLUSIONS

In the current study, despite track riders wearing protective equipment more often than recreational riders, there were no differences in injury severity or hospital outcomes between these two groups. Accordingly, this study suggested that motocross riders should not rely on protective equipment as the only measure of injury prevention. Additional safety measures are needed such as policy changes and increased enforcement of existing standards.

## Figures and Tables

**Table 1:** Comparison of demographics and hospital outcomes by treatment group.

	Total	Recreational Riders	Track Riders	P value
Number of Observations	115 (100%)	52 (45.2%)	63 (54.8%)	
Male Gender	108 (93.9%)	47 (90.4%)	61 (96.8%)	0.1505
Caucasian	112 (97.4%)	51 (98.1%)	61 (96.8%)	0.6752
Age Group				0.0959
Age 0 to 17	36 (31.3%)	11 (21.2%)	25 (39.7%)	
Age 18 to 54	74 (64.4%)	38 (73.1%)	36 (57.1%)	
Age 55 or older	5 (4.4%)	3 (5.8%)	2 (3.2%)	
ICU Admission, yes	27 (23.5%)	12 (23.1%)	15 (23.8%)	0.9265
Ventilation, yes	6 (5.2%)	3 (5.8%)	3 (4.8%)	0.8090
Hospital Disposition				0.5643
Home	104 (90.4%)	47 (90.4%)	57 (90.5%)	
Acute care or skilled nursing	1 (0.9%)	0 (0%)	1 (1.59%)	
Rehabilitation	9 (7.8%)	4 (7.7%)	5 (7.94%)	
Deaths	1 (0.9%)	1 (1.9%)	0 (0%)	

**Table 2:** Comparison of documented protective equipment status by treatment group.

	Total	Recreational Riders	Track Riders	P value
Number of Observations	115 (100%)	52 (45.2%)	62 (54.8%)	
Any Equipment, yes	84 (73%)	24 (46.2%)	60 (95.2%)	< 0.0001
Helmet, yes	84 (73%)	24 (46.2%)	60 (95.2%)	< 0.0001
Protective clothing, yes	56 (48.7%)	8 (15.4%)	48 (76.2%)	< 0.0001
Boots, yes	5 (4.4%)	0 (0%)	5 (7.9%)	0.0378
Neck, yes	4 (3.5%)	0 (0%)	4 (6.4%)	0.0644
Eyewear, yes	12 (10.4%)	0 (0%)	12 (19.1%)	0.0009

**Table 3:** Comparison of injury severity, age, and hospital outcomes based on protective equipment status by treatment group.

	Recreational Riders				Track Riders			
	Combined	No Protective Equipment	Protective Equipment	P value**	Combined	NoProtective Equipment	Protective Equipment	P value**
Number of Observations	52 (45.2%)	28 (53.8%)	24 (46.2%)		63 (54.8%)	3 (4.8%)	60 (95.2%)	
Injury Severity Score	7.7 ± 8.7*	7.3 ± 9.6*	8.3 ± 7.5*	0.6818	8.0 ± 6.08*	4 (3,4)†	8.3 ± 6.1*	<0.0001
Age	29.1 ± 14.0*	31.9 ± 13.8*	25.8 ± 13.9*	0.1142	23.8 ± 12.5*	21 (14,21)†	23.5 ± 12.2*	0.4109
Hospital Length of Stay	2.7 ± 3.1*	3.0 ± 3.8*	2.3 ± 2.0*	0.4229	3.1 ± 4.4*	2 (1,2)†	3.2 ± 4.5*	0.6575
ICU Admission, yes	12 (23.1%)	6 (21.4%)	6 (25%)	0.7606	15 (23.8%)	0	15 (25%)	NA
ICU Length of Stay	1 (1, 3.5)†	1 (1, 2)†	2 (1, 4)†	0.4712	1 (1, 3)†	NA	1 (1, 3)†	NA
Ventilation, yes	3 (5.8%)	3 (10.7%)	0	NA	3 (4.8%)	0	3 (5%)	NA
Ventilation Days	1 (1, 1)†	1 (1, 1)†	NA	NA	3 (3, 20)†	NA	13 (3, 20)†	NA

* All values were presented as mean ± SD.

** Calculation of ICU and ventilation days were based on those who utilized these services.

† All values presented as median (Q1, Q3) due to small number of cases.
